# An Account of a Remarkable Disease of the Heart, Lungs, and One of the External Mammæ; with the Morbid Appearances as They Presented on Dissection

**Published:** 1789

**Authors:** Robert Kinglake

**Affiliations:** Surgeon at Chipping-Norton in Oxfordshire


### LONDON MEDICAL JOURNAL.

I. *An Account of a remarkable Disease of the Heart, Lungs, and one of the external Mammæ; with the morbid Appearances as they presented on Dissection. Communicated in a Letter to Dr.* Simmons, *F. R. S. by Mr.* Robert Kinglake, *Surgeon at Chipping-Norton in Oxfordshire.*

EVERY endeavour to investigate the causes of disease deserves well of the community at large, but more particularly of medical practitioners; and indeed the latter appear unanimously anxious to observe every notable circumstance that occurs in their professional pursuits. It is a pleasing connexion to see the doctrine of distempers apply in explanation of the various dissimilar forms under which they are too often disguised; and, if possible, a still more valuable acquisition, when we can discover, by ocular testimony, what has eluded the researches of our closest reflection. Instances frequently happen where dissections prove the most plausible theories to have been been founded in error; the symptoms and dissection of the following case would seem, in some respects, to justify the truth of this remark.

Catherine Kinch, of Enstone, in the county of Oxford, aged twenty-one years, and of a very delicate habit of body, began, about three years since, to complain of an uneasy sense of motion at her heart, which she attributed to a jolt she had recently received when riding in a carriage. From this period may be dated the commencement of a disease that afterwards became a source of incessant affliction, and at length terminated in death.

The most predominant complaint for a considerable time, at least what chiefly engaged attention, was an enlargement of the whole substance of the left breast. This enlargement was not like that sort of induration which frequently occurs on the glandular structure of the breast, for it bore a nearer resemblance to hydropic tumours, feeling soft, and yielding to the touch; but did not, like anasarcous or œdematous tumefactions, retain an impression of the finger. What is more singular is, that from the whole cuticular surface of the breast was copiously emitted a fluid, differing from the formal appearances of purulent matter only in being lefs vifcid; and in proportion as this exfudation was more or lefs abundant would be an increafe or diminution of pain, both throughout the whole extent of the tumour, and in the cavity of the cheft, more particularly in the left fide about the region of the heart. Eafe, or rather a remiffion of pain, invariably enfued an increafe of the difcharge, and *vice verfa.*

Whatever caufe this morbid drain proceeded from, it greatly contributed to allay the irritation that prevailed on the tumid breaft and thoracic vifcera. After it had continued about fix months, the pain, tumour, and difcharge, began gradually to fubfide; and as the fymptoms became lefs confiderable at the breaft, the mucous glands of the pofterior fauces were more and more affected, until at length every fymptom that had marked the complaint at the breaft was now apparent in the throat, leaving no doubt of the affection having been transferred to this part.

No advantage accrued to the patient from the tranfpofition; on the contrary, the previous uneafinefs at the heart and lungs was aggravated thereby to a moft formidable throbbing, and fenfe of fuffocating ftricture, alternating nating with almoſt a ſtate of inaction and conſequent ſyncope.

The diſcharge from the fauces was not only troubleſome by keeping up a conſtant neceſſity for ſpitting, but it often acquired ſuch a degree of acrimony (probably from the circumſtances of confinement and heat) as to inflame and ſometimes excoriate the membrana faucium, rendering both ſpeaking and ſwallowing equally painful.

No cough at any time attended, unleſs when excited by the diſcharge happening to be unuſually glutinous, and tenaciouſly adhering to the uvula and tonſils, or when in cleaning the throat, by gargling, a ſmall quantity of the fluid eſcaped the epiglottis, and ſtimulated the trachea, ſo that its occurrence was purely accidental, and by no means characteriſtic of pulmonary affection.

The ſtate of the heart and lungs now ſeemed to claim the firſt attention. Medicine, tried in every form and variety that could be likely to render ſervice, availed nothing. Bleeding, which was at firſt uſed ſparingly, and at diſtant intervals, had but little better effect, until the extreme preſſure of the ſymptoms demanded its more frequent employ, and proved it to be the the only ſure means of leſſening the moſt exquiſite ſufferings, and obviating certain death. It was, therefore, repeated perhaps with an unprecedented frequency and continuance. A loſs of blood was found equally ſerviceable in moderating the violent palpitations of the heart, and reſtoring it to action when almoſt motionleſs.

During the ſpace of two years at leaſt three hundred and twelve venæſections were performed. About four ounces were the average quantity of blood taken away at a time; a leſs quantity was found by experience to have no effect. The operation was repeated at firſt twice a week, then every other day, and latterly every day. The relief given was uniformly the ſame. To deſcribe the benefit gained by each bleeding would be to exhibit the difference between the moſt afflicting pain and comparative eaſe. It immediately alleviated the ſeverity of every ſymptom, and, inſtead of deſtroying ſtrength, it ſenſibly invigorated the powers of the ſyſtem; but ſuch was the fatal complication of diſeaſe, that nothing more than temporary palliation could be obtained.

At length ſymptoms of general irritation took place, accompanied with irregular returns

 of of fever, under which the patient gradually funk, and died, on the 23d of May, 1789, in the moft tabid ftate imaginable.

The day after her deceafe I was permitted (in confequence of the patient's moft earneft requeft) to open the body, when many very interefting appearances of difeafe prefented.

The pericardium was found to contain a full pint of water; and both fides of the thorax were filled with a fimilar fluid, excepting where the cavity was obliterated by morbid adhefions to the pleura. At a fmall diftance beyond the finus venofus, a little within the anterior edge of the right auricle, were found clofely adhering to the fides of its cavity, and alfo to that of the right ventricle, feveral diftinct flefhy concretions refembling polypi, of different fizes. The largeft was as big as a common walnut, and fituated in the right auricle *. Thefe adventitious

* On careful examination I found thefe concretions were not of a perfect vafcular or mufcular ftructure; they were, for the moft part, exanguious; minute fibrillæ were indeed vifible, but fo thickly enveloped in an adipofe fubftance as to make but a very fmall proportion of their bulk: they were not unlike the defcription given by Thomas Bartholin in his Anatomy, where he fays, "Præter naturam multa in ven-
" triculis ventitious ſubſtances obſtructed more than half of the cavities of the right auricle and ventricle. In the trunk of the pulmonary artery, or rather in its tunics, about half an inch beyond the valvulæ ſigmoidales, was diſcovered a hard ſtony ſubſtance, weighing about half a drachm, and projecting ſo far into the cavity of the artery as very much to abridge its capacity. The lungs were not ulcerated, but the bronchial veſſels were univerſally loaded with a diſeaſed ſort of mucus. The abdominal viſcera were for the moſt part apparently ſound, excepting the ſtomach, which was very much extenuated in its natural ſubſtance, feeling more like a thin delicate membrane than a denſe muſcular organ. The glands and cutis of the affected breaſt were in a natural ſtate; neither had the tonſils, uvula, or neighbouring glands, ſuſtained

" triculis reperiuntur. Bauhinus fruſtula adipoſa & Wor-

" mius noſter experientiſſimus ex utroque ventriculo carun-

" culas quaſdam interius albicantes exterius rutilo colore

" emergentes quod & nos Patavii pridem & Haffniæ in diſſec-

" tionibus noſtris invenimus . . . Eraſtus concretionem pitui-

" toſam, flaveſcentis inſtar medullæ, quæ in boum coctis oſſi-

" bus reperitur; Veſalius glanduloſæ nigricantiſque carnis li-

" bras duas, Beniventus fruſtulum carnis inſtar meſpili." — Vide Th. Bartholini Anatome, 8vo. Lugd. Bat. 1673. p. 393.

a visible change: but what seems still more extraordinary is, that, notwithstanding the excessive evacuation of blood, the whole vascular system, even to the vasa vasorum, was rather full of coagulated blood than otherwise, and the muscles had not lost their natural redness.

These were the appearances which more particularly deserved attention, and which perhaps may be admitted in explanation of the various symptoms that prevailed. The external complaint of the breast had most probably a local consent with the internal irritation arising from the embarrassed action of the heart, as the latter greatly increased with the transference of the former. The extraordinary exsudation of the breast was not unlikely influenced by the general plethora produced by the polypose concretions in the right auricle and ventricle of the heart impeding a ready admission of blood from the venæ cavæ. The resistance was also added to by the calculus in the pulmonary artery *, and the fixed pain in the right lobe of the lungs may be reasonably ascribed to the ir-

* The patient had complained of a constant pain in her side for several years, nearly corresponding to the part where the stone was found.

ritation ritation of that foreign body. The watery collections would appear to be the natural confequence of weaknefs, obftruction, and redundancy.

The morbid excrefcences found in the cavity of the heart were moft likely the refult of inflammation, occafioned by the painful jolt before mentioned. The procefs by which inflammation produces new parts is too well underftood to require a particular explanation. It may be fufficient to obferve, that the exhaling veffels of the interior furface of the heart, as well as of any other part of an animal body, when duly ftimulated, will furnifh coagulable lymph capable (in convenient circumftances) of becoming a fit medium for vafcular extenfion and growth. Thus new parts are generated, the inflammation ceafes, and with it the creative action. A newly-formed fubftance, if adequately nourifhed, will permanently remain; if not, it will gradually wafte until it wholly difappears.

Although calculi are more frequently found in the courfe of the urinary paffages and biliary ducts, yet they may be formed in any part of the body, as we find (among many other inftances) from the peculiar fituation of the ftone alluded alluded to. Indeed this will not appear very extraordinary when we confider that the animal juices plentifully abound with calcareous matter, of which calculous fubftances in general are principally compofed. Either the red globules of the blood, coagulable lymph, or incipient offification, may become a nucleus or attracting point; but the fubfequent accretion and increafe are perhaps chiefly derived from calcareous earth *.

As no lefion of parts, or folution of continuity, as it is called, was to be feen either on the cutis of the breaft, or membrane of the fauces, the difcharge from thefe furfaces muft have been produced by an unhealthy action of found veffels; an additional inftance this in fup-

* Whatever theories were entertained by the ancients refpecting a new formation of parts were more or lefs embarraffed by the humoral pathology, and perplexed by the fuppofed falutary power of the vis medicatrix naturæ: a curious inftance of this occurs in Bartholin's explanation of preternatural fubftances — "Omnia ab humorum ficcitate & ad mo-
"tum tarditate in fenibus & ægris deduco. Utitur tamen hoc
"defectu natura ad tardiorem motum fanguinis incitandum
"& accelerandum, ficut aquæ injecto ligno facilius labuntur,
"vel ne fanguis totus grumefcat, ficut mulierculæ & laniones
"bacillo agitant fanguinem in ufus farciminis." — Vide Th. Bartholini Anatome, page 394.

port port of the doctrine that a denuded ſurface is not a *ſine qua non* to the formation of purulent matter. I believe this theory is now pretty generally admitted, and the attentive obſerver will perhaps invariably find that the fluid which reſults from a deranged action of ſound parts will aſſume more or leſs of the appearances of pus, according as the inflammatory action has been exceſſive, moderate, or deficient. A certain force or extent of action is neceſſary to produce a genuine ſuppuration; and any conſiderable deviation from this ſtandard will be ſhewn by a correſponding variation from its perfect ſtate.

That the vaſcular ſyſtem was not more depleniſhed of its blood might be owing to the digeſtive faculties of the ſtomach having acquired an increaſed aſſimilatory power to ſupply the deficiency that muſt have been induced by the frequent loſs of blood, on the principle of inanition being a ſtimulus to the animal œconomy to furniſh what is wanting. The ſame law of nature extends to all the viſceral functions of the body. Thus if the ſtomach and bowels are empty, the ſenſation of hunger is excited; if the urinary bladder, the kidnies are ſtimulated; if the ſeminal veſſels, an increaſed creafed fecretion of femen follows; and what is more applicable, if the gall bladder and biliary ducts are emptied by vomiting, the fecreting action of the liver will be fo far augmented as to fend an amazing redundancy of bile to the duodenum; and why, on parity of reafon, may not the conftitution at large, *pro tempore*, call on the digeftive organs (as the natural medium through which it receives its nourifhment) for that fupport which the exigences of its exhaufting ftate may require. Thus has provident nature fo fecured a due execution of the office of life as to make even irregularities operate as remedies. The organic derangements found in this cafe were too locally fixed to admit of a curative indication being drawn from them in fimilar circumftances; yet they may be allowed fome claim to attention both in a phyfiological and pathological point of view. What, for inftance, can be more interefting than to obferve nature ftruggling againft obftacles to the circulation feated at the very fountain of life; or to attend to the advantages of an unparalleled feries of blood letting, which feemed to be effected by diminifhing the impetus of the circulation from the venæ cavæ into the right auricle? And when we confider the difeafed fubftance in the cavities of the heart, the calculus of the lungs, the fingular affection of the left breaft, and its metaftafis to the throat, the hydropic collections and morbid adhefions (all of which are circumftances more or lefs deferving of notice) we cannot but acknowledge, that although they might be in themfelves irremediable, yet that there is fomething more than unprofitable fpeculation, or mere theoretical amufement, in well diftinguifhing what caufes of difeafe are neceffarily fatal, and why they are beyond the reach of medical art.

*Chipping-Norton,*

Auguft 13, 1789.

